# Measuring the effectiveness of management interventions at regional scales by integrating ecological monitoring and modelling

**DOI:** 10.1002/ps.4759

**Published:** 2017-11-23

**Authors:** Robert P Freckleton, Helen L Hicks, David Comont, Laura Crook, Richard Hull, Paul Neve, Dylan Z Childs

**Affiliations:** ^1^ Department of Animal & Plant Sciences University of Sheffield Sheffield UK; ^2^ Department of Biointeractions and Crop Protection Rothamsted Research Harpenden UK

**Keywords:** density‐structured model, vector generalized additive model, integrated weed management, population model, weed ecology

## Abstract

**BACKGROUND:**

Because of site‐specific effects and outcomes, it is often difficult to know whether a management strategy for the control of pests has worked or not. Population dynamics of pests are typically spatially and temporally variable. Moreover, interventions at the scale of individual fields or farms are essentially unreplicated experiments; a decrease in a target population following management cannot safely be interpreted as success because, for example, it might simply be a poor year for that species. Here, we argue that if large‐scale data are available, population models can be used to measure outcomes against the prevailing mean and variance. We apply this approach to the problem of rotational management of the weed Alopecurus myosuroides.

**RESULTS:**

We derived density‐structured population models for a set of fields that were not subject to rotational management (continuous winter wheat) and another group that were (rotated into spring barley to control A. myosuroides). We used these models to construct means and variances of the outcomes of management for given starting conditions, and to conduct transient growth analysis. We show that, overall, this management strategy is successful in reducing densities of weeds, albeit with considerable variance. However, we also show that one variant (rotation to spring barley along with variable sowing) shows little evidence for additional control.

**CONCLUSION:**

Our results suggest that rotational strategies can be effective in the control of this weed, but also that strategies require careful evaluation against a background of spatiotemporal variation. © 2017 The Authors. *Pest Management Science* published by John Wiley & Sons Ltd on behalf of Society of Chemical Industry.

## 1 INTRODUCTION

In agro‐ecology a major challenge is to be able to predict the outcome of management interventions.^1^–^3^ Given the growing requirement for more efficient use of land and resources,^4^ there is a pressing need to use these more efficiently as well as to reduce the environmental impact of food production.^5^ One of the limitations of optimizing management at all scales is in measuring the effectiveness of different options.^6^ In an ideal world, experimental trials can be used to assess new methods for management and subsequently deployed in the field. However, given the spatial and temporal variability of the real world, the impacts of management in the field may be difficult to assess.^7^

Arable weeds are a worldwide problem for crop production and are costly in terms of yield reductions, in addition to the financial costs of the machinery and chemicals needed to control them.^8^–^10^ Moreover, there are indirect costs associated with weeds^11^; for example, some rotational or management combinations are not possible when weeds are an established problem. As an example, minimum tillage may be limited by the occurrence of sterile brome (*Anisantha sterilis*)^12^ or continuous winter wheat is limited by build‐up of populations of *Alopecurus myosuroides* (HL Hicks *et al*., unpublished). Globally, weed problems are exacerbated by the widescale evolution of herbicide resistance in many cropping systems, including within those reliant on genetically modified herbicide‐tolerant crops.^13^

At the scale of an individual population (e.g. a population of weeds in a field), the problem of accounting for spatiotemporal variability becomes especially difficult with many species showing extreme variation over space or time.^14^–^16^ This variability has consequences for interpreting outcomes.^17^ For example, a manager might deal with an emerging problem in a given year by using one or several different interventions. In the following year, a decrease in the numbers might be observed, leading to the conclusion that management was successful. However, this conclusion might be premature if other factors simultaneously change.^18^ For example, if the year happened to be a poor one for all populations, then the reduction in density would be observed across all fields whether the management interventions were applied or not and, not knowing this wider context, the manager would mistakenly attribute the reduction to the intervention applied.

Even if an intervention is applied successfully, a second problem concerns long‐term management outcomes.^19^ Although a reduction in the densities of a pest might be observed in one season, the question of whether long‐term reduction in densities can be achieved is not certain. For example, ploughing can reduce population sizes of weeds from one season to the next by burial of seed below a depth from which they can successfully germinate and emerge.^20^–^22^ However, applying the same management in successive years will be ineffective in reducing densities because this will simply return seed from deep burial to the surface leading to an eventual stable equilibrium.^20^

Population models are predictive tools that can be used to forecast future densities.^23^ They can be used to simulate how different management options will affect population sizes.^24^ Models take a range of forms from simple mathematical models^25^–^27^ to complex process‐based simulations.^28^ Models for weed populations have been developed for many species and deployed for a suite of purposes.^26^, ^29^–^31^ These include models that simulate the consequences of different types of management,^28^ the effects of introducing genetically modified herbicide‐tolerant crops,^32^ and the effects of climate change on future distributions.^33^ Such successes clearly demonstrate the utility of models in a range of situations. However, the use of models for management at the field or farm level has been limited. In large part this is because developing location‐specific local‐scale models for population dynamics is extremely difficult for almost any ecological population, not exclusively arable weeds.

One of the limitations of models is that they are typically data hungry: data are needed to parameterize or validate, ideally with information available across spatially and temporally replicated populations subject to different management regimes.^23^, ^24^ This is true of even very simple models. However, such data are rarely available and hence models have been limited in the extent to which they can be deployed at large scales.^34^ Indeed, it has been argued that models are essentially limited in their utility for weed ecology because of the likely predominant influence of local and site‐specific factors.^34^, ^35^

Here, we deploy weed population models in a novel way for determining the success or not of management interventions. It has been noted previously that local‐scale applications of population models is extremely difficult owing to local variations.^35^, ^36^ Here, we propose that this problem can be overcome when coarse‐grained, but informative data can be obtained across large numbers of populations. When we have data on large numbers of populations subject to the same management, spatiotemporal variation in population dynamics can be measured and the range of variation in dynamics can be quantified. This variation effectively sets a baseline distribution against which the consequences of alternative management can be compared, and we propose that such benchmarking can be enormously informative in understanding management outcomes.

In this paper, we use models of fields subject to alternative management to quantify the range of variation in responses across different populations. This is the largest scale application of weed population models to our knowledge. For each population, we fit a model to characterize population dynamics and from this derive an overall variance in the expected outcome of different management. Against this variance of management outcomes, we compare the population dynamics of alternative interventions to ask whether there is evidence that this management affects populations against the background range of variation we would normally expect to see. We show that combining large amounts of data, population models and local context data allows us to effectively measure the impacts of alternative management options.

## 2 METHODS

### 2.1 Study system

We studied the grass weed blackgrass (*A. myosuroides*) over two seasons in 2014 and 2015. In 2014, some 70 farms were surveyed across the lowland arable region of the UK. Sites were chosen to represent a range of farming management typical of the region. At each site, two fields of winter wheat were chosen, one field estimated to contain the highest densities of weeds on the field, the other chosen to contain the lowest.

To address the problem of generating data at sufficiently large scales, we have developed density‐structured approaches for monitoring and modelling population dynamics.^37^–^39^ This is an empirically focused method of data modelling that is built upon rapid density monitoring. Based on relatively large survey units (e.g. 20 × 20 m for arable weeds) and ordinal density state assessment (e.g. low, medium, high, very high), this approach can be used to survey extensive areas very rapidly. This permits large amounts of data to be collected in a short period. Importantly, this allows many fields to be censused during a single field season. Based on such surveys, repeated in successive years, the dynamics of populations can be potentially studied under a wide range of management conditions.

Using this approach, surveys were conducted by a team of three observers. Prior to surveying, a GPS system was used to create a system of quadrats of size 20 × 20 m across the field. Typically, fields contained ∼ 100–200 quadrats, comprising an area of up to 8 ha per field. For fields smaller than 8 ha, we sampled the field by choosing a single contiguous area within the field at random. Our intention was to capture within‐field spatial pattern as well as generate measures of incidence, hence we chose to sample contiguous areas.

Following the methodology described in Queenborough *et al*.,^38^ fields were walked to estimate densities of blackgrass in each of the quadrats. The densities of weeds within each quadrat were assigned to one of five ordinal density categories (zero, low, medium, high and very high). As outlined in Queenborough *et al*.,^38^ these categories were chosen based on previous datasets, and the assignments of states are highly repeatable both by and between observers. This approach is a rapid survey methodology designed to provide field‐scale data across multiple fields. As such it its necessarily a compromise between precision and extent. Simulation results show that models derived from these coarsened ordinal data are capable of accurately representing the ‘true’ population dynamics.^38^ There is potentially some reduction in precision at the tails of the distribution of population sizes: the impact of this is minimized by using previous survey data to inform the choice of density states, for example, as in Freckleton *et al*.^37^

The current analysis focuses on two management options at two scales. The first concerns a rotational change across a suite of farms, the second is changing within field management on single farm.

### 2.2 Changing field management

One management recommendation that has been widely adopted is that farmers switch from growing winter wheat to growing spring crops, especially spring barley, in order to manage *A. myosuroides*. In our sample, we observed 12 fields making a transition from winter wheat in 2014 to being sown with spring barley in 2015. We therefore focus on this sample of fields to assess the efficacy of this control method, relative to those fields that remained in winter wheat (22 fields).

### 2.3 Local within‐field management

The second management type of intervention we evaluated was a change in in‐field management in response to escalating problems with blackgrass. One farm manager responded to high blackgrass densities by sowing areas of high and very high density with higher than conventional densities of barley seed (∼ 50% higher). The objective we wished to address was whether we could use models to assess whether this management shows evidence of effectiveness. In principle, we are restricted in our ability to do this because the management intervention is in effect an unreplicated experiment and flawed from a statistical perspective. However, the reality of real‐world management is that this situation arises continually, with farmers making interventions in such a manner. Our assertion was that models could provide a tool to help interpret such data by reference to other fields and farms.

### 2.4 Modelling

The model is based on a Markovian transition matrix describing the change in states of quadrats from one census to the next. The state variable at time *t* is a vector of proportions of quadrats in each state *i* at time t, *s_i_*(*t*), i.e.

(1) $$N\left(t\right)={\left\{s_{0}\left(t\right),s_{L}\left(t\right),s_{M}\left(t\right),s_{H}\left(t\right),s_{V}\left(t\right)\right\}}^{T}$$

The subscripts O, L, M, H and V denote absent, low, medium, high and very high, respectively. The state at the next time step is modelled using a state transition matrix, **T**, the entries of which are *p_ji_* is the probability that a quadrat in state *i* at time *t* is in state *j* at time *t* + 1:

(2) $$T=\left[\begin{array}{ccccc} p_{00} & p_{0L} & p_{0M} & p_{0H} & p_{0V}\\ p_{L0} & p_{\mathrm{LL}} & p_{\mathrm{LM}} & p_{\mathrm{LH}} & p_{\mathrm{LV}}\\ p_{M0} & p_{\mathrm{ML}} & p_{\mathrm{MM}} & p_{\mathrm{MH}} & p_{\mathrm{MV}}\\ p_{H0} & p_{\mathrm{HL}} & p_{\mathrm{HM}} & p_{\mathrm{HH}} & p_{\mathrm{HV}}\\ p_{V0} & p_{\mathrm{VL}} & p_{\mathrm{VM}} & p_{\mathrm{VH}} & p_{\mathrm{VV}} \end{array}\right]$$

The model for the state value at the next time step is then:

(3) $$N\left(t+1\right)=\mathrm{TN}\left(t\right)$$

This is a simple linear Markov model for the state changes.^37^ The matrix **T** summarizes all of the process operating within the population to influence population numbers, including competition, density‐dependence and seed bank dynamics.

Because **T** summarizes state transitions, with the number of quadrats being conserved from one generation to the next, the rows of **T** therefore sum to 1. Consequently, the dominant eigenvalue of **T** is always 1. Insights into the impacts of changing the elements of **T** on population dynamics may thus be obtained from analysing the second eigenvalue of **T**. Specifically the damping ratio, *ρ*, measures the ratio of the second to the first eigenvalues. Larger values of this imply that perturbations from the stable state distribution of the eventual equilibrium persist longer, hence smaller values imply ‘faster’ dynamics.

In the current context **T** describes a transition between states in different environments (i.e. winter wheat and spring barley). Equation (3) then may more properly be written as:

(4a) $$N_{S}\left(t+1\right)=T_{\mathrm{SW}}N_{W}\left(t\right)$$

(4b) $$N_{W}\left(t+1\right)=T_{\mathrm{WW}}N_{W}\left(t\right)$$

where the subscripts *S* and *W*, denote spring barley and wheat, respectively. Note that in equation (4) the transition matrices are field‐specific. These represent only partial models for the system because we do not currently have data on the transitions among states for populations making transitions from spring barley to winter wheat.

### 2.5 Model fitting

We fitted equation (4) to our data using Vector Generalized Linear Models (VGLMs).^38^, ^40^ VGLMs are a flexible class of Generalized Linear Models that permit a range of models to be fitted to data. In the current application, we used VGLMs because they allowed us to model discrete data using multinomial distributions.

The statistical model fitted for the transition to state *j* in a given field (*k*) from state *i* in the previous year is:

(5) $$\text{log}\left(\frac{p_{\mathrm{ji}}}{p_{\mathrm{Vi}}}\right)=a_{\mathrm{ij}}+b_{\mathrm{jk}}$$

The response variable is a log‐odds expressing the log probability of making the transition to state *j* relative to the probability of making the transition to the maximal state, state *V*. This normalization is because, in general, for *S* states there are *S* – 1 free parameters that can be estimated. *a* is an intercept term, i.e. measures the probability for the average field, whereas *b* is an additional parameter to model the field‐specific effect in field *k*. Note that equation (2) is simpler than the maximal model which would be:

(6) $$\text{log}\left(\frac{p_{\mathrm{ji}}}{p_{\mathrm{Vi}}}\right)=a_{\mathrm{ij}}+b_{\mathrm{ijk}}$$

Unfortunately, equation (6) can be fitted only when there are sufficient data to estimate all pairwise transitions in equation (2). However, this is not the case unless sample sizes are very large because some transitions are not observed or occur in low numbers. VGLMs, like other linear modelling approaches, model deviations from an overall average through estimating an effect for each field. Thus, for transitions with small or absent samples, robust parameter estimates are generated through a field effect. In equation (2) this takes the form of a field level effect on transitions from each state.

### 2.6 Statistical methods

The data on which our surveys are based are ordinal categorical density states. To compare field densities between years and management systems we converted the individual estimates to integers (0–4) and calculated a simple mean density. Although coarse, this is a pragmatic approach and justified by the approximately logarithmic nature of the density scale that we use.^38^

To analyse the variance in model outcomes we used a transient Life Table Response (tLTRE).^41^ This is based on analysing one‐step ahead dynamics accounting of variation in both population structure and intrinsic dynamics. We analysed the outputs of the models by firstly generating models for each field, i.e. 22 matrices for transitions from winter wheat to winter wheat, and 12 for transitions from winter wheat to spring barley. We then applied each of the models to the initial densities of *A. myosuroides* from each field, i.e. 34 matrices applied to each of the 34 fields, yielding 34 × 34 = 1156 combinations. Using the change in density of *A. myosuroides* as a response variable, we employed a linear mixed effects model to estimate the variance components associated with the field (i.e. the location being modelled), the matrix (the source of the matrix applied) and the rotation (i.e. continuous winter wheat versus rotation between winter wheat and spring barley).

Scripts and datasets for running the analyses reported in this paper are available from: https://figshare.com/s/39c6a4868c4558f5dbcc .

## 3 RESULTS

### 3.1 Overall effects of management on densities

**Figure 1:**
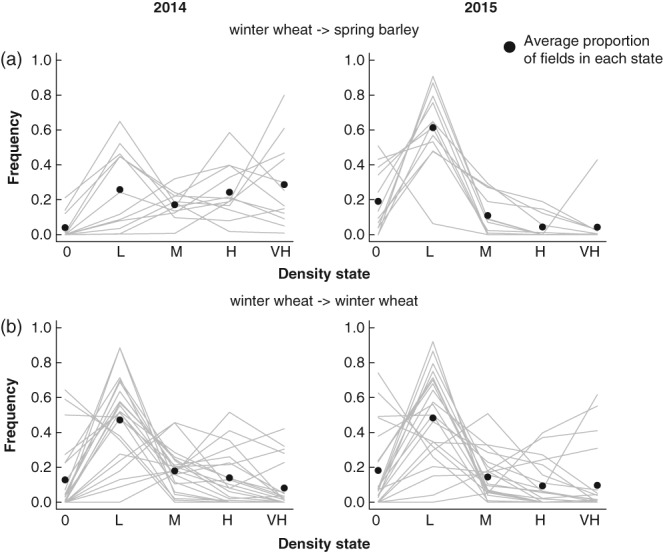
Densities of Alopecurus myosuroides in fields with contrasting rotational management in successive years. Density states within 20 × 20 m quadrats are measured on an ordinal scale (0, absent; L, low; M, medium; H, high; VH, very high; see Methods for details) across whole fields (see Fig. 2 for examples). The proportion of such quadrats in each state in each field is shown. Grey points show the average proportions across all fields. (a) Fields rotated from winter wheat to spring barley between 2014 and 2015. (b) Fields maintained in winter wheat between 2014 and 2015.

**Figure 2:**
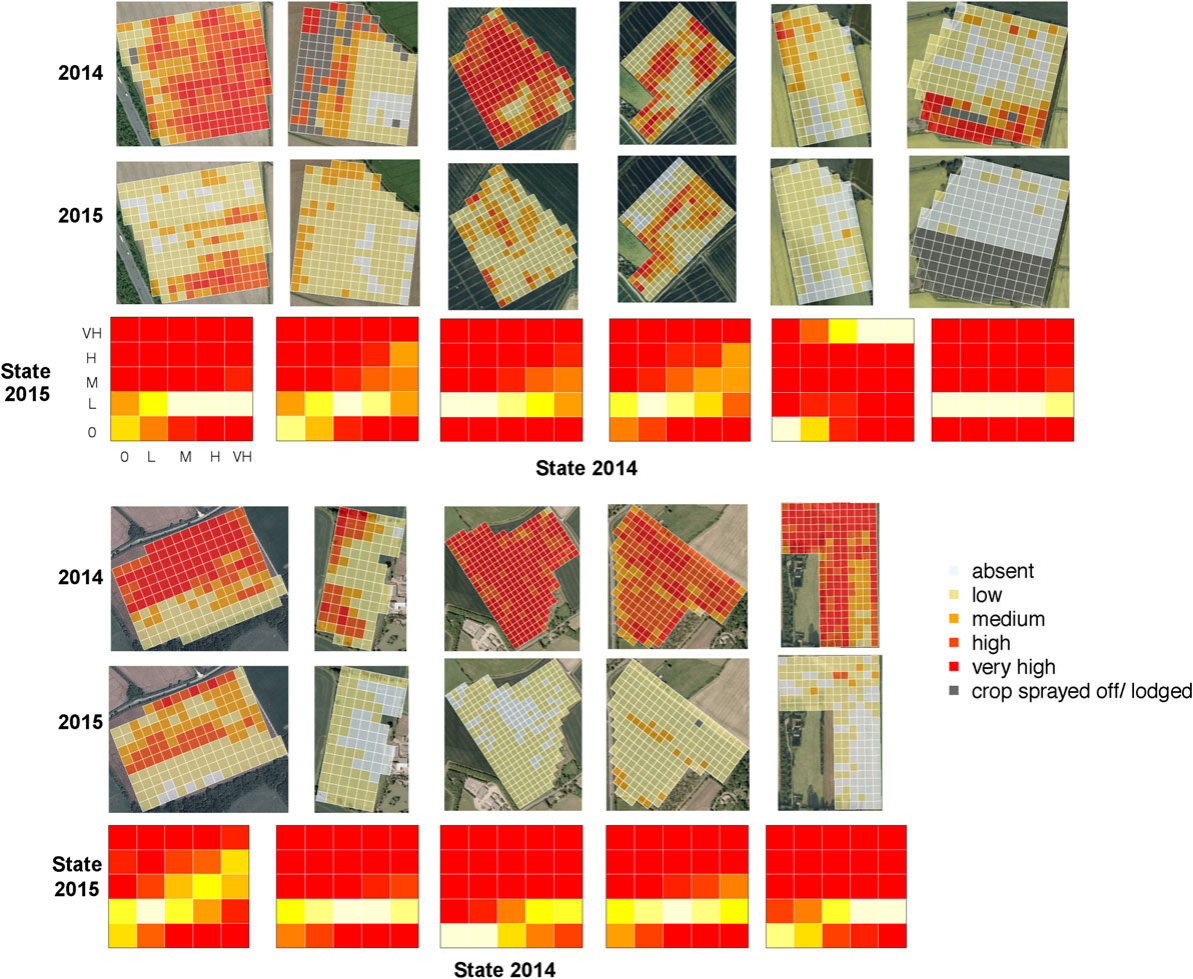
Examples of field‐scale distributions of weeds in successive years, together with fitted transition matrices (see text for details of the fitting method). All fields shown were rotated from winter wheat to spring barley between 2014 and 2015. The transition matrices are represented as heat maps (red, low probability; yellow and white, high probability).

Fields that were rotated into spring barley had lower densities of *A. myosuroides* in 2014 (Fig. 1a), compared with the densities in 2014 of those which were maintained in winter wheat (Fig. 1b; Welch's *t* = 3.00, df = 21.89, *P* = 0.007). Examples of the distributions of weeds in field before and after management are shown in Fig. 2. As shown in Fig. 1a, fields that were rotated into spring barley showed an approximately increasing distribution of densities, with fields dominated by medium, high and very high density plots (Fig. 1a). By contrast, the fields that remained in winter wheat were dominated by low density plots (Fig. 1b). Overall this probably reflects farmer behaviour: farmers were choosing to rotate into spring barley those fields with high densities of *A. myosuroides*.

**Figure 3:**
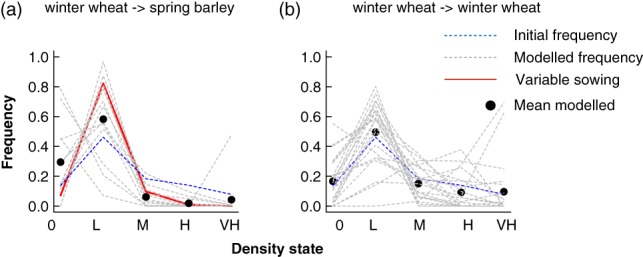
Modelled responses of populations of Alopecurus myosuroides to rotational management. In these examples, the starting condition was the 2014 distribution of density states of the average field which were maintained in winter wheat (blue dashed line). Each line represents a matrix generated from a different field under the two forms of management. Thus, these responses measure: (a) what would have been the density of an average non‐rotated field had it been planted with spring barley compared with (b) the predicted response from maintaining winter wheat. The red line in (a) represents the field that was managed with variable sowing densities.

**Figure 4:**
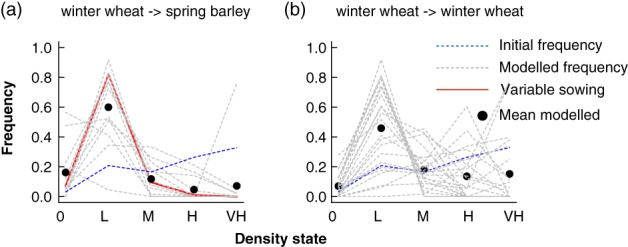
Modelled responses of populations of Alopecurus myosuroides to rotational management. In these examples, the starting condition was the 2014 distribution of density states of the average field rotated from winter wheat to spring barley (blue dashed line). Each line represents a matrix generated from a different field under the two forms of management. Thus, these responses measure: (a) what would have been the density of an average non‐rotated field had it been planted with spring barley compared with (b) the predicted response from maintaining winter wheat. The red line in (a) represents the field that was managed with variable sowing densities.

**Figure 5:**
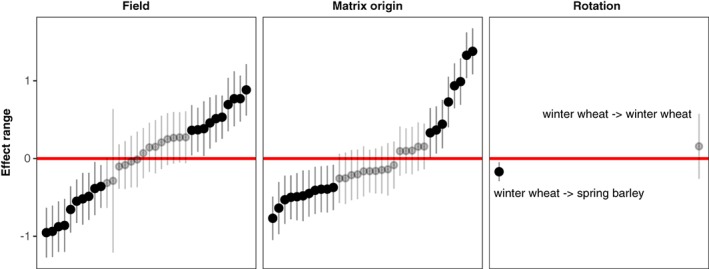
Analysis of the sources of variation in modelled outcomes of population dynamics. We generated models for each of 34 fields, 22 of which were maintained in winter wheat, 12 rotated from winter wheat to spring barley. We applied each model to the initial density state. The response variable was change in population size and we used a linear mixed model to estimate variance components due to three sources: ‘Field’, the initial state in each field; ‘Matrix origin’ the location from which the transition matrix model was estimated; and ‘Rotation’ the sequence of crops from one year to the next. The effect range is the estimate of the random effect for each field, location or rotation. We used a parametric bootstrap to estimate confidence intervals. Grey points have confidence intervals that overlap zero.

By contrast, in 2015 the difference was cancelled with no significant difference between densities in those fields rotated into spring barley and those maintained in winter wheat (Fig. 1; Welch's *t* = 1.32, df = 31.45, *P* = 0.20). Both sets of fields were dominated by low densities in 2015. This very likely reflects that farmers were using crop rotation as part of their strategy to manage populations of *A. myosuroides*. We calculated the change in population size (*N*(*t* + 1) – *N*(t)) and found that this was significantly lower in fields rotated to spring barley than those maintained in winter wheat (*t* = 3.52, df = 22.46, *P* = 0.002).

### 3.2 Local effects versus inter‐annual trends

One of the problems with evaluating the impacts of management is that local trends can be confounded by broad‐scale inter‐annual variations, as well as difficult to interpret in the face of variation within fields. Our large‐scale data allows us to test this; we calculated the difference between densities of *A. myosuroides* in successive years and tested whether this differed between rotations using a linear model. We report two sets of transient growth analyses in which the initial conditions were varied (Figs 3 and 4) and then summarize the results of the full tLTRE in Fig. 5.

In the first set of simulations (Fig. 3), the initial state was set as the average state of fields that remained in winter wheat between years (Fig. 1b). Figure 3 contrasts the outcome of applying matrices from rotated fields (Fig. 3a) with that of applying the matrices from unrotated ones (Fig. 3b). The models predict that rotation from winter wheat to barley led to slightly lowered densities, particularly by reduction of the higher density states (Fig. 3a). On the other hand, the models for fields remaining in winter wheat predict much more variable outcomes: although the modal state is the ‘low’ one, the proportions of quadrats predicted to be in higher states increases, as does the apparent variability of the outcome. As shown in Fig. 5, the effect on the density of *A. myosuroides* of rotating to spring barley is statistically significant, although marginally so, reflecting the pattern evident in Fig. 3(a) that there is considerable spatial variation.

The initial state in the second set of simulations was the same as the average (Fig. 4) state of those fields that were rotated to spring barley, i.e. with a larger proportion of sites in the higher density states (Fig. 1b). For this initial starting condition, the difference between the models for the two management options is even clearer. The rotation from winter wheat to spring barley results in a clear reduction in the densities of the highest states relative to the starting condition (Fig. 4a), whereas average densities of *A. myosuroides* are predicted to be much higher when fields are not rotated (Fig. 4b). The combined results in Figs 3 and 4 suggest that the strategy of rotating from winter wheat to spring barley is successful in reducing densities, relative to not rotating at all. Nevertheless, there is considerable variation in the outcome between fields.

The degree of spatial variation in the outcome of management is shown in Fig. 5. This summarizes the variance in change in the density of *A. myosuroides* resulting from differences in initial conditions (field: field‐to‐field variation in initial density), the model (i.e. field‐specific transition matrix from which each individual projection is derived) as well as the rotation (continuous cropping versus rotating with spring barley). Based on a Linear Mixed Model (LMM) we estimated variance components of 0.279, 0.263 and 0.053 for these components, respectively, together with a residual variance of 0.407. As is clear in Fig. 5, this reflects similar levels of variation in initial conditions and matrix origin on the outcome, with the rotation contributing a smaller amount to the overall variance. In all, the variance components from the LMM suggest that the combination of field specific factors (initial conditions and field‐specific transitions) contribute 54% of the variance in modelled outcome, with rotation explaining only ∼ 5%. Of course, in reality this is the only aspect of the system over which the farmer has control, but there is a wider question about whether this expected 5% effect is sufficient to warrant any costs involved in changing rotation.

#### 3.2.1 Assessing the effects of within‐field management

As described above, a single field was subject to variable crop sowing density in which the areas of the field with high and very high densities of *A. myosuroides* were sown with an increased density of spring barley. In this field there was a significant reduction in weed density between these two years (paired *t* = 8.53; df = 195; *P* < 10^−14^) indicating that management was successful in reducing weed densities.

Against the background of huge variation in population dynamics between fields, we found no clear evidence in our analysis to indicate that the dynamics within this field were different from other fields cropped with spring barley. Specifically, the observed distribution of densities within the field was well within the range of values for other fields, irrespective of starting density (Figs 3a and 4a).

## 4 DISCUSSION

A challenge for ecology is to keep pace with advances in technology. In agriculture we are at the point at which it is possible to routinely collect large‐scale data on a suite of aspects of farm management.^42^ Tools such as low‐cost GPS‐enabled machinery,^43^ together with equipment that permits routine monitoring of yields, crop quality and soil conditions,^44^ generate a huge amount of context data. The approaches we have developed for modelling and monitoring weed populations are designed to generate correspondingly large datasets on field‐scale distributions. In the future the development of Unmanned Aerial Systems (UAS) technology is likely to further extend both the scale and grain at which we can collect data.^45^, ^46^ Moreover, the results we obtain showing that outcomes are spatially and temporally enormously variable, indicate that even detailed local‐scale studies such as long‐term trials need to be integrated into the wider context.

### 4.1 What to do with so much data?

Although data availability is increasing at an enormous rate, the question of what to do with such data is less obvious. A clear problem is that much readily‐collected data is essentially retrospective in nature: yields are measured when the crop has ceased to grow; weeds are typically measured once they are large enough to be visible. In the case of *A. myosuroides*, plants are most visible when the seed heads are ripening, and this is too late to prevent seed return. Given that weed density data are likely to be available too late to inform management in the current growing season, the question is how can this information be used to inform future management. We argue that the answer to this is to combine benchmarking of outcomes with prospective population models.

### 4.2 Benchmarking outcomes

Because management outcomes are likely to be temporally and spatially variable in effectiveness (Fig. 1),^36^ we advocate benchmarking management outcomes against distributions of intervention effects based on large amounts of existing data. Figure 1 illustrates this idea, crudely. Using comparisons of densities of weeds from large numbers of fields, we can do two things. First, it is possible to evaluate whether, on average, the management intervention is effective or not. Second, because the variance in outcome is measured, the effectiveness within any individual farm or field can be evaluated relative to the overall distribution.

The value of modelling in benchmarking is twofold. The first contribution of models is in enabling the observed outcomes to be measured as a dynamic process. This leads to the second important use of models in benchmarking, which is to explore the importance of initial conditions for measuring management outcomes. We found that fields which were rotated to spring barley from winter wheat initially had higher densities of weeds than those which were maintained in winter wheat. This is an empirical observation and an example of the value large‐scale data in analysing management. Not recognizing this initial bias in management could affect both observational and modelling analysis of the effectiveness of future management.

The modelling analyses presented in Figs 3 and 4 are ‘virtual experiments’ that explore projected outcomes as if the initial conditions could be varied. Such analyses also allow the effects of inter‐annual variations to be accounted for, for instance the possibility that densities were reduced in all fields because conditions were poorer in the second year. Modelling analyses go beyond simple comparisons of densities in permitting us to explore the impacts of a range of factors.

We were able to analyse the data from a single field to determine whether there was evidence that additional management in this field (variable sowing density) was more effective. Our results indicated that the outcome of management was not obviously different from what we observed in other fields. Consequently, we do not have compelling evidence that this management was effective.

We found that in 2015 the densities of weeds were not significantly different between fields cropped with spring barley and those containing winter wheat. This emphasizes that a dynamic context is important in understanding the outcomes of management. In this case, the initial weed densities were higher in those fields sown with spring barley. Looking forward, our models are currently unable to forecast whether the reductions in weed density are likely to be maintained because we do not yet have data on weed density transitions in fields that are rotated from spring barley to winter wheat or other crops.

Forecasts using such models and data will be essential in deciding on long‐term management outcomes. As noted above, some management options may only yield transient benefits. Ploughing, for instance, will provide effective short‐term control of *A. myosuroides* 47; however, the dormancy of seeds of this species^48^ means that burying seed is not a long‐term management strategy for reducing densities. Combining data and models allows us to evaluate such outcomes.

### 4.3 Importance and value of modelling

It is impractical to create bespoke ecological models for local populations that can be used predictively.^24^ This is because local conditions are likely to vary considerably from site‐to‐site, as are the influences of historical and landscape factors that also are likely to have considerable influence on population dynamics of weeds.^49^ Our data support this view, with the outcome of either of the management options considered here (rotating to spring barley or maintaining winter wheat) being variable at the field scale (Fig. 1). For example, in the sample of fields that we measured, at least one field maintained an extensive (∼ 40%) coverage of very high density weed infestation following a switch to spring barley (Fig. 1a), contrasting with most other fields which showed substantial reductions of infestations of this level. In general, the outcome of management is best regarded as a distribution of responses and management predictions needed to reflect both the mean and the variance in the response, as well as historic factors.

### 4.4 Implications for control of A. myosuroides

Growing spring barley as a method to control *A. myosuroides* is effective for two reasons. First, the germination profile of *A. myosuroides* peaks in the autumn, so that a high proportion of seed germinates between September and December.^50^ Subsequent flushes of germination occur, which can generate additional cohorts of weeds, particularly when the soil is disturbed.^51^ However, seed‐bed preparation prior to the spring sowing of barley removes most of the early‐emerging cohorts, reducing the number of *A. myosuroides* plants that can develop through to maturity.

The second reason for spring barley's effective control of *A. myosuroides* is due to its greater competitiveness compared with other cereals such as wheat. The competitiveness of barley is driven by rapid accumulation of height and biomass,^52^ effective in suppressing establishing *A. myosuroides* plants. Because *A. myosuroides* is typically autumn germinating, the growing season is also effectively reduced in spring barley crops. This adds to the effectiveness of control of *A. myosuroides* in spring crops in general.

It has long been appreciated that increasing crop density can aid in the control of weeds through competition.^53^ The relationship between yield of both crops and weeds and densities is well known to be characterized by reciprocal linear functions.^54^, ^55^ The potential for increasing sowing density to increase crop competitiveness and hence contribute to weed control is well grounded in theory, but the implementation requires tuning and understanding of the relationship between crop competition and weed population dynamics.

## 5 CONCLUSION

Population dynamics are variable at almost any scale at which we study them. This is a challenge for making predictions in applied ecology. Here, we tackled this problem head‐on through attempting to quantify the variation in population dynamics at an unprecedented scale. We have shown that, having quantified the variance in population dynamics, the outcomes of management can be interpreted through benchmarking relative to the average, and integrating across the variance. Moreover, using models it is possible to conduct virtual experiments that allow variations in initial conditions to be controlled for. In the specific case study, we have shown that the overall management strategy is successful in the short term, but that there is limited evidence for success of a variant. We hope that the simplicity of the empirical and analytical framework will permit future applications in this and other species.
